# Personalized Evaluation of Atrial Complexity of Patients Undergoing Atrial Fibrillation Ablation: A Clinical Computational Study

**DOI:** 10.3390/biology10090838

**Published:** 2021-08-28

**Authors:** Ana María Sánchez de la Nava, Ana González Mansilla, Esteban González-Torrecilla, Pablo Ávila, Tomás Datino, Javier Bermejo, Ángel Arenal, Francisco Fernández-Avilés, Felipe Atienza

**Affiliations:** 1 Department of Cardiology, Instituto de Investigación Sanitaria Gregorio Marañón (IISGM), Hospital General Universitario Gregorio Marañón, 28009 Madrid, Spain; ansan38a@doctor.upv.es (A.M.S.d.l.N.); agmansilla@salud.madrid.org (A.G.M.); etorrecilla@telfonica.net (E.G.-T.); pablo.avila@salud.madrid.org (P.Á.); tomas.datino@salud.madrid.org (T.D.); Javier.bermejo@salud.madrid.org (J.B.); arenal@secardiologia.es (Á.A.); Francisco.fernandezaviles@salud.madrid.org (F.F.-A.); 2 CIBERCV, Centro de Investigación Biomédica en Red de Enfermedades Cardiovasculares, 28029 Madrid, Spain; 3 ITACA Institute, Universitat Politécnica de València, 46022 València, Spain; 4 Facultad de Medicina, Universidad Complutense de Madrid, 28040 Madrid, Spain

**Keywords:** atrial fibrillation, computational models, ablation, personalized models

## Abstract

**Simple Summary:**

Atrial fibrillation is a type of arrhythmia that occurs when the electrical activity of the heart in the atrium is not coordinated, and its consequences can be lethal. The driving source that initiates this chaotic activity can be located anywhere in the atrium, but most frequently appears in certain areas such as the pulmonary veins. In this study, we developed a new estimation method to evaluate possible source location and complexity of the arrhythmia using computer simulations. This method represents mathematical descriptions of natural processes that can be used to mimic a real scenario, including specific information such as the atrial anatomy. Here, we identified a specific biomarker the enabled obtaining a foci distribution map and found that elimination of pulmonary vein drivers was associated with a successful long-term ablation outcome. This study could, therefore, help to identify and characterize patients in order to better plan the ablation procedure.

**Abstract:**

Current clinical guidelines establish Pulmonary Vein (PV) isolation as the indicated treatment for Atrial Fibrillation (AF). However, AF can also be triggered or sustained due to atrial drivers located elsewhere in the atria. We designed a new simulation workflow based on personalized computer simulations to characterize AF complexity of patients undergoing PV ablation, validated with non-invasive electrocardiographic imaging and evaluated at one year after ablation. We included 30 patients using atrial anatomies segmented from MRI and simulated an automata model for the electrical modelling, consisting of three states (resting, excited and refractory). In total, 100 different scenarios were simulated per anatomy varying rotor number and location. The 3 states were calibrated with Koivumaki action potential, entropy maps were obtained from the electrograms and compared with ECGi for each patient to analyze PV isolation outcome. The completion of the workflow indicated that successful AF ablation occurred in patients with rotors mainly located at the PV antrum, while unsuccessful procedures presented greater number of driving sites outside the PV area. The number of rotors attached to the PV was significantly higher in patients with favorable long-term ablation outcome (1-year freedom from AF: 1.61 ± 0.21 vs. AF recurrence: 1.40 ± 0.20; *p*-value = 0.018). The presented workflow could improve patient stratification for PV ablation by screening the complexity of the atria.

## 1. Introduction

Atrial Fibrillation (AF) is the most common arrhythmia, affecting a total population of 33.5 million worldwide [1]. Circumferential pulmonary vein isolation (CPVI) is considered the standard therapy for symptomatic AF patients [2]. However, non-pulmonary vein drivers located at the posterior wall, superior vena cava, the interatrial septum sites, the terminal crest or the coronary sinus can be found and are responsible in part for the inefficiency of the ablation procedure, especially in persistent AF patients [3].

Ablation planning and evaluation of atrial pro-arrhythmic behavior may play a key role toward ablation outcome. For this purpose, the combination of personalized atrial models implemented with electrophysiological and anatomical biomarkers of abnormal behavior have been integrated in a simulation environment to help identify arrhythmic behavior and improve novel diagnostic [4] and treatment strategies [5,6,7,8,9]. Computational simulations have emerged in this field as a new tool that can be used for characterization and prediction in different scenarios, from prediction of cardiotoxic compounds [10,11], targeted ablation [12] or recurrence after ablation [13].

In this field, automata models have been used for electrophysiological simulations to achieve simpler approaches with lowered computational time as compared to other models that include ionic level description. A lowered computational cost is translated into faster simulations with a higher number of possibilities to explore [14].

Here, we present a novel methodology to predict the efficacy of AF ablation based on computer simulations that included patient anatomy and different arrhythmic scenarios (i.e., different rotor location and number). These simulations were later compared with the clinical results of patients undergoing electrocardiographic imaging (ECGi) maps, CPVI and 1-year ablation outcome.

## 2. Materials and Methods

### 2.1. Patient Database

We included patients undergoing CPVI for drug-refractory paroxysmal (*N* = 14, 9 female) and persistent AF (*N* = 16, 8 female). Candidates were patients >18-years old, history of symptomatic AF, included if sustained AF was inducible during the electrophysiological study. Patients included in this study were admitted for ablation of drug-refractory paroxysmal and persistent AF, undergoing circumferential point-by-point ablation [2]. All patients gave informed consent. The protocol was approved by the institutional review board of the Hospital General Universitario Gregorio Marañón.

### 2.2. Atrial Electroanatomical Complexity Evaluation Protocol

Atrial electroanatomical complexity was evaluated analyzing the number and distribution of AF reentrant sites in relation to the anatomic characterization of the atrium. To that purpose: (1) MRI imaging from patients were obtained; (2) computational simulations of cardiac activity in the reconstructed atrium were performed; and (3) the results of simulations were compared with patients’ clinical characteristics, ECGi complexity and outcomes after ablation.

**Figure 1:**
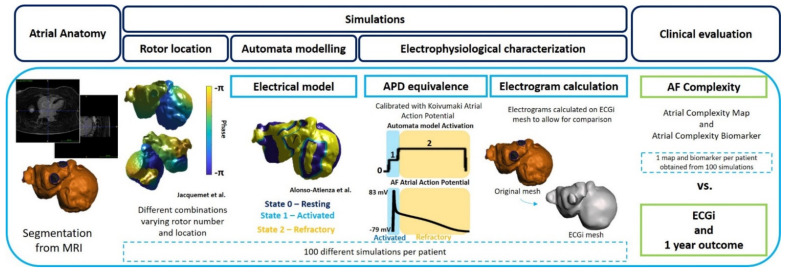
Simulation protocol (from left to right): Biatrial anatomy segmentation from MRI. Rotor location with Jacquemet algorithm on atrial anatomy. Simulation with 3-state protocol (Alonso Atienza et al. model) with rotor location obtained from Jacquemet et al. protocol. Electrophysiological characterization by translation of the activation pattern into APD equivalence and later electrogram calculation. Evaluation of the simulation by means of new biomarker calculation and validation with rotor histogram.

The workflow followed for the evaluation of atrial pro-arrhythmic behavior is explained below and summarized in Figure 1.

#### 2.2.1. Atrial Anatomy

Magnetic Resonance Imaging (MRI) was performed in all patients before ablation procedure. MRI images with a spatial resolution of 0.7 mm × 0.7 mm × 1.5 mm were acquired 2–3 days prior to the ablation procedure and segmented using ITK-SNAP [15]. Images were segmented to obtain a 3D mesh of both atrial cavities using growing region automatic segmentation for both atria separately. Later, Meshmixer software was used to combine both left and right atrium. After obtaining the raw anatomies, meshes were resampled to obtain a 200 µm resolution for simulations. An example of the final mesh used for simulations, which includes the atrial anatomical complexity present in patients, can be observed in Figure 1. Left atrial area (mm^2^) was measured in every anatomy for further analysis together with the electrophysiological variables obtained from the workflow.

#### 2.2.2. Computational Models of the Atria

Once the atrial anatomies were segmented, simulations were run under AF conditions where rotational activity could be characterized. Overall, for each anatomy, 100 simulations per patient run with different initiation protocols using the corresponding individualized anatomical model for 1000 ms. These simulations had an arbitrary location of rotational activity patterns on the atrial cavity ranging from 1 to 10 rotors simultaneously, ensuring the total coverage of the atria for later evaluation.

This protocol was repeated 10 times per geometry to increase the number of simulations, achieving a final number of 100 simulations per anatomical model. First, rotational activity was distributed over both cavities of the atria with different locations each time. After the location of the rotors was obtained [16], the automata model was run to evaluate the evolution of the scenario and posterior characterization. These models, despite having simpler formulations, allow for the presence of more complex scenarios, including location of higher number of rotors. The models for rotor location and activation model are explained in subsequent sections. A brief description of the difference between ionic-level electrophysiological models and automata models showing examples in 2D planes is further discussed in Supplementary Material (Figure S1).

##### AF Initiation: Automatic Rotor Location

Jacquemet et al. algorithm [16] was implemented for the development of automatic location of the rotational activity. This implementation, based on an eikonal-diffusion solver, allows obtaining computed activation maps similar to those obtained in the mono-domain model, with the option of varying the number of rotors in the model from 1 single rotational foci up to 14 [16]. The location of the rotational activity was arbitrary and only depended on the curvature of the model. As Jacquemet’s algorithm is calibrated in phase, a conversion into the labels for the automata model (3 discrete states) was performed to continue with the workflow. An example of this implementation can be found in Figure S2 together with the characterization of the simulations (Figure S3).

##### Automata Model Simulations

An automata model based on activation patterns was implemented to simulate cardiac activity in the atrial cavity. Alonso-Atienza’s model [14] was implemented to perform simulations with three different states (state 0 or resting, state 1 or activated, and state 2 or refractory period) that depended on the probability equation depicted below, where E represents the excitability of the unit, A the activation and D the distance matrix.

(1) $$P_{j}^{exc}=E*Q=E*\;\underset{I\neq J}{\sum }\frac{A_{i}}{D_{ij}^{2}}$$

A more detailed description of this model, including all the equations and variables, can be found in the Supplementary Material. All nodes in the mesh were simulated following this model, i.e., no differences were implemented for different regions nor fiber orientation. NVIDIA Titan XP was used for all the simulations and posterior analysis of the workflow. Simulations were run in Microsoft Visual Studio 2017 and characterization of the simulations was performed in Matlab. The estimated ionic simulated model cost was 275 min vs. automata model: 42 min for 1-second simulation during AF, including stabilization and arrhythmia induction for the ionic model.

##### Electrophysiological Equivalence and Characterization

The evaluation of the electrophysiological properties of the simulations, which included the 3 states of the simulations of the automata, were calibrated using Koviumaki Action Potential Duration [17] to translate the automata model into measurable atrial electrophysiological signals. For this purpose, the square pulses that are identified as activations in the automata model, were directly substituted with the atrial APD morphology.

Once the electrophysiological information was recovered, electrograms were calculated for each node. More specifically, from each simulation, a uniform mesh of pseudo-unipolar electrograms was calculated under the assumption of a homogeneous, unbounded, and quasi-static medium [18]. The mesh used for the electrogram calculation was individualized and corresponded to the same mesh used for the ECGi calculation, allowing a direct comparison between both analyses.

In addition, the logarithmic energy entropy, which has been extensively used for the characterization of signals in other disciplines [19], as well as for cardiac signals [20], was calculated on the electrograms for each node and normalized for each atrial anatomy. More specifically, this entropy showed similar performance in prediction algorithms in previous studies [20] as Shannon entropy, widely used in the electrophysiological field. Finally, the mean entropy of the electrograms from all the simulations for a given patient was calculated and evaluated using entropy maps.

The main output of the workflow was produced by means of Atrial Complexity Maps (ACM) and Atrial Complexity Biomarker (ACB). ACM were obtained from the average entropy values of all the simulations from a given patient. ACB was obtained from the quantification of the number of rotors attached to the PV in the sustained simulations for each patient, which were later averaged. A rotor was considered to be attached if rotational activity was maintained around the PV for the complete simulation.

#### 2.2.3. Clinical Evaluation

##### AF Complexity: Atrial Complexity Map vs. ECGi

**Figure 2:**
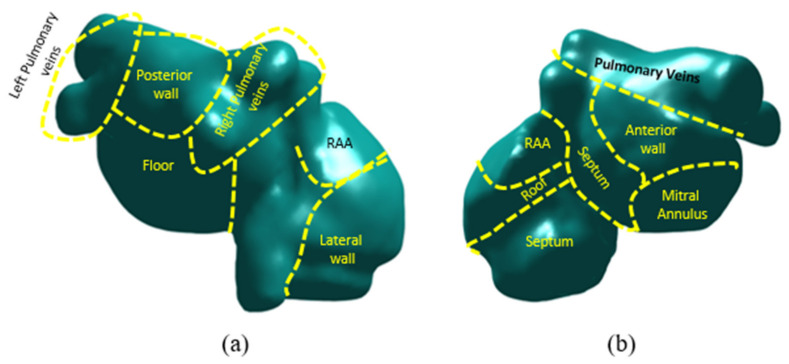
Division of the atria for rotor and entropy maps coincidence evaluation. (**a**): posterior view of atrial anatomy. (**b**): anterior view of atrial anatomy.

We compared the number of AF simulations with maintained reentries (ACM) obtained from the simulation workflow with the histogram of rotors obtained from the ECGi calculation. As explained in previous sections, the entropy maps were calculated with the same anatomies that the ECGi for them to be comparable. The specific protocol for obtaining and calculating ECGi was previously described [4,21,22]. Briefly, a minimum of three segments of at least 1 s duration were selected to calculate the histogram of rotors from ECGi signals. Rotors were obtained by counting the number of rotors in each atrial model node from the ECGi calculations for each of the segments. Finally, all three histograms were averaged and compared with the results of the simulations. Comparison was performed by dividing the complete anatomy by areas as shown in previous studies [23] and in Figure 2, and evaluating the presence of rotors and high entropy foci per area. This methodology enabled the characterization and evaluation of the complexity of the atria.

##### AF Complexity and 1-Year Ablation Outcome

Evaluation of the simulations was performed attending to the number of sustained simulated AF episodes per patient, rotors attachment to the pulmonary vein (ACB) and left atrial appendage, rotor distribution between both cavities, mean conduction velocity, and maintenance of more complex scenarios. These parameters were evaluated as predictors of ablation efficacy at 1 year. Additionally, AF type (Paroxysmal vs. Persistent) was also compared to reveal possible characterization patterns using the workflow.

#### 2.2.4. Statistical Analysis

The *t*-test was used to evaluate the statistical significance between continuous paired or unpaired variables, and statistical significance was considered for *p* < 0.05 for continuous variables. Pearson Chi Independence test was used to evaluate categorical or binary variables, and statistical significance was considered for *p* < 0.05. All data are reported as mean ± SD. In addition, a regression analysis to test the independent predictive value has been conducted including the following parameters: AF type, gender, simulations results and 1-year outcome.

## 3. Results

### 3.1. Cohort Description

In total, 30 patients were included in this retrospective study. The characteristics of patients included in the study are shown in Table 1. When patients were compared according to 1-year post-ablation outcome, there were no significant differences in age, height or weight. In contrast, the proportion of persistent AF patients at 1 year was significantly higher on the AF group and the same trend was observed for female patients.

### 3.2. Comparison of ACM with ECGI

**Figure 3:**
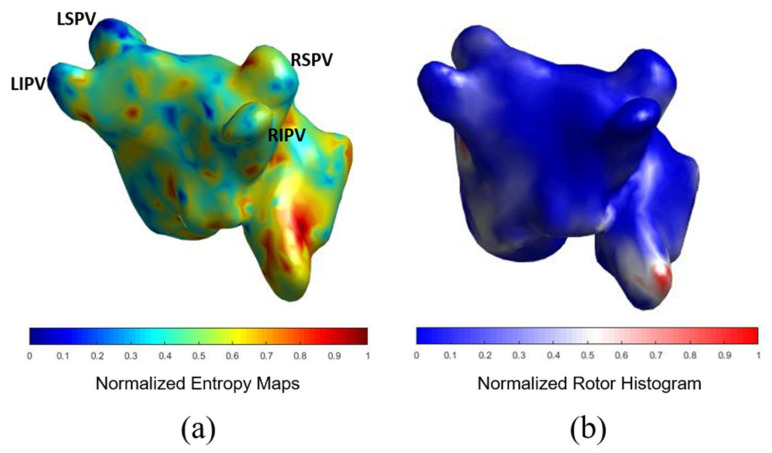
Comparison of simulations entropy maps with histogram of rotors in sample case. (**a**) shows the results for the computational method. (**b**) shows the histogram of rotors computed from the ECGI.

**Figure 4:**
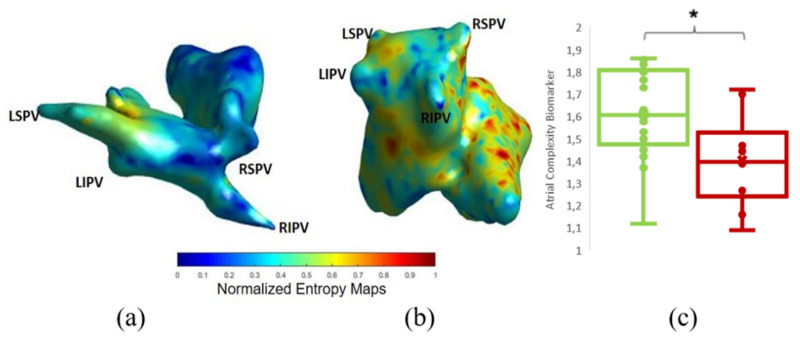
Example of normalized entropy maps for (**a**) Successful and (**b**) Unsuccessful ablation. (**a**) shows higher entropy on the pulmonary vein area while (**b**) shows a more diffused distribution of high entropy areas. (**c**) showed the ACM or mean number of rotors in simulations around the pulmonary vein area for AF freedom (green) and AF cases (red). * *p* < 0.001.

In Figure 3 we show the coincidence between the histograms of rotors recorded in patients ECGi and high entropy areas obtained from the simulation protocol. Atrial Complexity Maps were identified as descriptors of the atrial complexity, where the characteristics observed on the histogram of rotors showed a direct correlation with 1-year post ablation outcome. This correlation showed 93.33% similarity in the pulmonary vein area and posterior wall, 80% coincidence in the floor area, 86.67% in the lateral wall and 83.33% in the right atrial appendage. An example of a simple Atrial Complexity Map is shown in Figure 4a where the entropy foci is mainly located on the left superior PV occurring in a patient that maintained SR at 1-year after ablation. Figure 4b shows a heterogeneous and complex Atrial Complexity Map with multiple high entropy foci, i.e., for electrograms that presented entropy values higher than 0.8*maximum entropy, distributed on both atria, with low rotor attachment to the PV area, which occurred in a patient with AF recurrence during follow-up.

### 3.3. AF Complexity and 1-Year Ablation Outcome

#### Sustained AF Simulation Induction and Rotor Distribution

**Figure 5:**
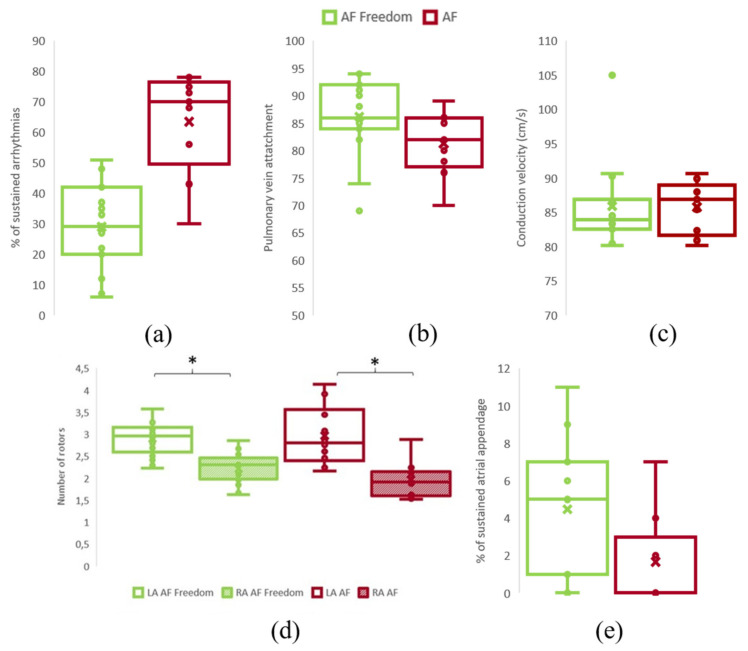
Simulation characterization. (**a**). Percentage of sustained F in both groups., (**b**). Percentage of simulations of rotors with pulmonary vein attachment in both groups. (**c**). Conduction velocity of simulated scenarios. (**d**). Number of rotors in the LA and RA for both groups. (**e**). Percentage of rotors with left atrial appendage attachment in both groups. * *p* < 0.001.

There was no difference in the number of induced sustained AF episodes during the simulations in relation to 1-year ablation outcome (freedom of AF: 33.00 ± 17.82 vs. AF: 34.90 ± 17.63 simulations; *p*-value = 0.79) (Figure 5a). All 30 anatomies presented more than 70% attachment of at least one rotor to the PV area in the simulations, independently of the group (freedom of AF: 86.20 ± 7.06 vs. AF: 81.33 ± 5.97 simulations; *p*-value = 0.10) (Figure 5b).

The percentage of patients that presented high entropy values on the pulmonary vein area on the ACM was significantly higher on the freedom of AF group (*n* = 18) than of the AF group (*n* = 12) (freedom of AF: 93.75% vs. AF: 62.5%; *p* < 0.001), supporting the favorable ablation outcome in the former group. Moreover, freedom of AF patients presented lower number of high entropy areas or simpler ACM on the RA than AF patients (freedom of AF: 68.75% vs. AF: 100%; *p* < 0.001).

Patients with freedom of AF at 1-year presented higher values of the Atrial Complexity Biomarker (i.e., a higher number of rotors attached to the PV) than patients with AF recurrence during follow-up (freedom of AF: 1.61 ± 0.21 vs. AF: 1.40 ± 0.20; *p*-value = 0.018) (Figure 4c). Interestingly, the mean number of sustained left atrial appendage rotors tended to be higher on the group of AF Freedom (freedom of AF: 3.52 ± 3.81%; AF: 1.50 ± 2.37%; *p*-value: 0.14) (Figure 5e). From the complete set of simulations, the number of rotors was higher on the left atrium (LA) than in the right atrium (RA) for both groups: AF Freedom patients (LA: 2.96 ± 0.35; RA: 2.22 ± 0.34; *p*-value < 0.0001) and AF patients (LA: 2.97 ±0.68; RA: 1.97 ±0.40; *p*-value < 0.0001) (Figure 5d). Differences in mean conduction velocity during AF were not significantly different between groups (freedom of AF: 85.91 ± 6.18 vs. AF: 85.73 ± 3.81 cm/s; *p*-value = 0.94) (Figure 5c). From all the possible simulated scenarios, some presented more stability in time, that is, a higher percentage of simulations sustained AF during 1000 ms. The relationship between the number of initiated rotors with respect to the number of rotors maintained in the simulations is shown in Supplemental Figure S3. In this case, the average number of sustained AF simulations presented a decreasing trend for an increasing number of initiated SP, that is, the arrhythmia was not easily sustained for high number of simulated rotors.

Furthermore, there were no significant differences between the freedom of AF and AF group, except for the case in which the number of initiated rotors was fixed to two, in which the average number of maintained simulations of AF patients was higher in the AF group (freedom of AF: 3.87 ± 2.17 simulations vs. AF: 5.89 ± 2.09 simulations; *p*-value = 0.035). Further comparison of the electrophysiological description of the simulations can be found in Table 2.

### 3.4. Comparison with AF Type

There were significant differences in long-term outcome after ablation depending on the duration of AF (i.e., AF type) (Table 1). Four biomarkers were significantly different when paroxysmal and persistent patients were compared. Paroxysmal AF patients presented a higher number of sustained rotors (Paroxysmal AF: 5.30 ± 0.53; Persistent AF: 4.69 ± 0.56; *p*-value: 0.012), especially on the LA (Paroxysmal AF: 3.10 ± 0.41; Persistent AF: 2.65 ± 0.37; *p*-value: 0.01) and the PV antrum (Paroxysmal AF: 1.48 ± 0.32; Persistent AF: 1.14 ± 0.18; *p*-value: 0.006), with the number of sustained rotor attachment to the PV being higher on the paroxysmal group (Paroxysmal AF: 1.61 ± 0.18; Persistent AF: 1.42 ± 0.25; *p*-value: 0.02). In addition, results did not show any correlation with LA area, (Paroxysmal AF: 31.88 ± 7.20 cm^2^; Persistent AF: 30.95 ± 7.56 cm2; *p*-value: 0.74).

Regression analysis results show that the ACM biomarker is the only variable with a trend for independently predicting 1-year post ablation outcome (*p*-value = 0.0752) as compared to other clinical variables such as AF type (*p*-value: 0.2548) and gender (0.3442).

### 3.5. Applicability to Clinical Environment

**Figure 6:**
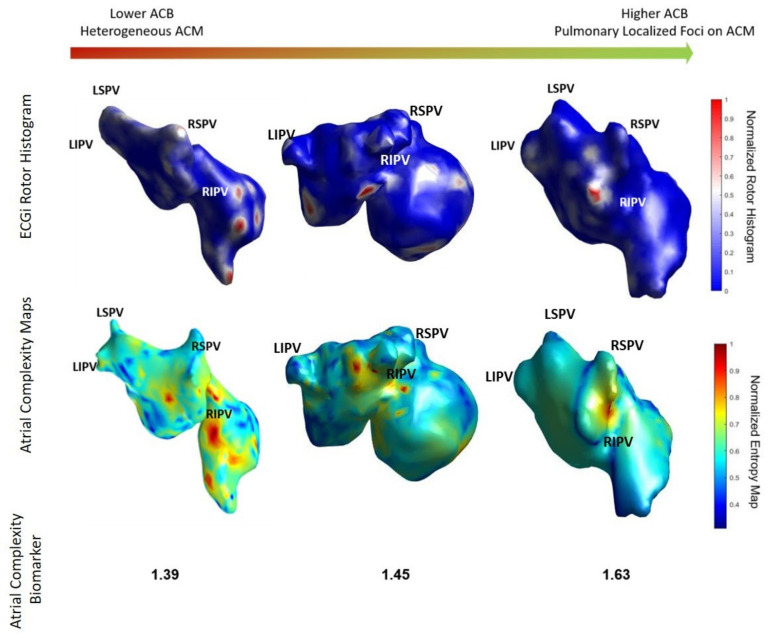
Rotor map of three different patients included in the study. Rotor maps with higher complexity are correlated with lower CAN and more heterogeneous ACM while simpler rotor maps are correlated with higher ACB and localized pulmonary foci on the ACM. ACM: Atrial Complexity Maps; ACB: Atrial Complexity Biomarker.

This methodology, and specifically, the ACM and ACB obtained from the workflow, are presented as an estimator of the complexity of the atrial activity. Figure 6 shows the rotor maps from three different patients ordered for increasing Atrial Complexity Biomarker values. As it can be observed, the higher the ACB, the lower the complexity of the rotor map with more localized the rotors appear on the pulmonary vein area. From left to right, patients with lower ACB presented higher number of rotors than patients with higher Atrial Complexity Biomarker. In addition, rotors in patients with low ACB were mainly located in both atria, whereas patients with high ACB presented the rotors concentrated on the PV area.

Examples of these cases are present in Figure 6, where the left panel, corresponding to an ACB with 1.39, represents a patient in which the ablation was not successful and both the ECGi rotor histogram and the Atrial Complexity Map present high activity foci on the right atrium. In contrast, the patient on the right panel represents a case in which the ablation was successful and both the ECGi rotor histogram and the Atrial Complexity Map present high activity foci on the right pulmonary veins, accompanied by a higher ACB. Overall, 11 patients (AF Freedom: 2 patients vs. AF: 9 patients) corresponded to an ACB lower than 1.45 and 19 patients (AF Freedom: 16 patients vs. AF: 3 patients) corresponded to an ACB higher than 1.50.

## 4. Discussion

This study presents a new simulation workflow for the personalized electrophysiological evaluation of the atria in a simulation environment. This is a proof-of-concept study that establishes a noninvasive evaluation of atrial electrophysiological complexity by means of two novel biomarkers: Atrial Complexity Maps and the Atrial Complexity Biomarker to evaluate atrial complexity and predict the efficacy of the ablation. Our results revealed that long-term successful AF ablation occurred in patients with rotors mainly located in the pulmonary veins, while unsuccessful procedures presented greater number of entropy foci outside the PV areas. Paroxysmal AF patients presented a significantly higher number of LA rotors with greater attachment to the PV area and lower density of entropy areas in the RA, as compared to persistent AF patients, explaining the improved long-term clinical outcome in the former group. These results are in agreement with clinical studies that consistently report a better outcome of the ablation following PV electrical isolation, while AF recurrences were most common in patients with multiple drivers distributed at extra-PV sites. [2,3,21,24,25].

### 4.1. Simulation Models

Detailed ionic models for the evaluation of AF induction at different scales include complementary information that may be relevant for the ablation procedure [11,26]. These ionic models also appear as a good approach for very specific workflows, such as pharmacological studies, which analyze the playing role of ionic channels and its modification using different drugs. In addition, several studies explore different strategies for the evaluation of the atrial complexity, including specific ablation strategies and targets [5], cycle length evaluation [27] and proarrhythmic structures such as the left atrial appendage [28]. However, they may present significant drawbacks, especially for large-scale simulations, due to the high computational power needed for such specific models, limiting the overall number of scenarios to be studied [29], therefore limiting the number of scenarios to be studied or the number of structures (i.e., only including left atrium). In contrast, simpler models, such as the automata model used in this study, can be applied for modeling an initiated arrhythmia behavior enabling the analyses of several distributions of rotational foci. Moreover, automata models rely on simpler activation patterns, and can be implemented and used on cardiac modeling to obtain similar approaches with a lower computational cost [14,30]. Therefore, the use of simpler models together with graphical processing units for parallel computation, reduces the total computational time, allowing a potential translation and implementation of this methodology in the clinical environment for patient evaluation.

These simulations are presented, as a workbench for characterizing the proarrhythmicity based on the anatomy and different arrhythmic scenarios. One of the main challenges in computation is the initiation of rotational activity on the desired area. Several approaches have been implemented and described in previous publications in order to tailor arrhythmia initiation by including remodeling such as repolarization alternants, adipose tissue modeling, and cardiac ion channel mutations [8]. However, we gave priority to the analysis of scenarios with different combinations of rotational activity that reflect the heterogeneity of the arrhythmias using an algorithm that directly deal with different rotors over the atria, comparing their different distributions. The inclusion of such a high number of scenarios or combinations of rotational foci (i.e., 100 simulations per anatomy) enables to include all possible areas at which rotors can be maintained, differing from other approaches in which a low number of combinations is analyzed, restricting the arrhythmic simulations to the pulmonary vein area and excluding the arrhythmia initiation on right atrium [31].

Regarding the characterization of the simulations, all simulated atria presented realistic models in which the number of rotors was higher on the left atrium than in the right atrium, with a similar number of maintained simulations per group and high attachment of rotational drivers to the pulmonary vein area, identified as the main proarrhythmic trigger on clinical practice. These results align with previous studies that reflect the dominance of the LA in the rotational activity of AF patients [4,24,25,32,33,34,35], demonstrating the reproduction of a clinical scenario into personalized simulations in a computer.

### 4.2. Clinical Implications

**Figure 7:**
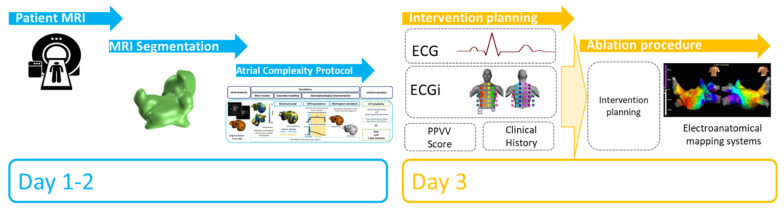
Protocol proposal for the integration of the method in a clinical environment.

The increasing number of potential candidates for ablation therapies is much higher than the availability of laboratories to perform procedures, but patients are selected based on very simple and unproved selection criteria for efficacy. However, current indiscriminate application of ablative therapies to large, unselected cohorts of patients with atrial fibrillation might dilute the intended treatment benefits and significantly increase the cost. Translation of the mechanistic insights of computational and basic research into clinical management concepts will uncover the full potential of personalized atrial fibrillation management [36]. The present approach may help appropriately select patients undergoing invasive therapies by: (1) integrating the workup protocol as shown in Figure 7, where anatomical characterization and simulations will be performed 2 days prior to the procedure, to later correlate high entropy areas location in the simulations protocol with ECGi, and help decide the ablation strategy; (2) giving preference for the standard ablation procedures to those patients with favorable predictors for ablation long-term success (low ACM, high ACB) [37]; and (3) selecting patients with higher atrial complexity to undergo the elimination of extrapulmonary drivers ablation [21,32,38].

This protocol is based on a personalized simulation method that could be potentially modified to input other remodeling factors such as fiber orientation to develop more complex fibrotic models, broadening the application to models closer to the clinical setting. Therefore, the integration of image-based computational modeling into treatments for heart rhythm disorders could thus advance personalized approaches to heart disease.

### 4.3. Study Limitations

The main advantage of the model—its simplicity—also constitutes its main limitation, as this model is not as tailored as ionic models. Furthermore, other scenarios such as different conduction velocity areas or fiber orientation should be implemented on the automata model and considered to study a wider population of patients. Second, we were able to demonstrate that all the areas that presented high-frequency activation on the ECGi presented high entropy values, but we were unable to ensure that all the high entropy areas were coregistered with high-frequency areas or rotational activity. Further studies should be conducted to evaluate if this mismatch is due to a lack of more ECGi episodes or if rotors were present only in part of the atrial anatomy.

In addition, other tailored characteristics such as fibrosis distribution over the atria and ionic heterogeneity [39] should be considered in further studies to better represent the anatomical and electrical remodeling of the cardiac tissue. Atrial thickness and blood pressure are two important factors that have been demonstrated to affect frequency dynamics and should be further explored to complement the models [40].

Finally, higher attachment to the left atrial appendage was observed on the AF freedom group, which exclusively underwent PV isolation. Further studies should confirm the proarrhythmic behavior of the left atrial appendage in these models [41].

## 5. Conclusions

This study presents a new method for the evaluation of the pro-arrhythmic areas on atrial anatomies providing Atrial Complexity Maps and the Atrial Complexity Biomarker as estimators of atrial complexity. This approach, validated using ECGi to measure atrial complexity, was able to identify the set of patients that presented higher atrial complexity.

## Figures and Tables

**Table 1:** Patients’ clinical characteristics and univariate analysis for 1-year outcome after ablation. Significant *p*-values can be found in bold.

Characteristics	Complete Cohort	AF-Freedom Group	AF Group	*p*-Value
Anthropometrics	30 patients	18 patients	12 patients	
Persistent AF	16 (53.3%)	7 (38.89%)	9 (75%)	**<0.001**
Age, yrs	59 ± 14	57 ± 15	62 ± 12	0.38
Female	17 (56.67%)	9 (50%)	8 (66.67%)	**<0.001**
Height (cm)	164.15 ± 9.38	164.87 ± 8.85	163.25 ± 10.33	0.67
Weight (kg)	76.03 ± 16.24	77.80 ± 14.51	73.67 ± 18.68	0.52
Blood samples				
Potassium	4.07 ± 0.40	4.04 ± 0.44	4.12 ± 0.35	0.60
Creatinine	0.91 ± 0.18	0.91 ± 0.17	0.90 ± 0.20	0.90
Hemoglobin	13.83 ± 1.67	13.57 ± 1.93	14.21 ± 1.18	0.32
Leucocytes	7.24 ± 2.29	7.65 ± 2.73	6.65 ± 1.34	0.25
Platelets	206.83 ± 47.90	212.94 ± 54.50	198.17 ± 37.16	0.42
INR	1.25 ± 0.55	1.22 ± 0.55	1.28 ± 0.57	0.80
LVEF	54.42 ± 9.67	53.00 ± 11.21	56.78 ± 6.24	0.37
Atria Size (cm^2^)	31.49 ± 7.88	30.71 ± 8.83	32.87 ± 6.05	0.52
Previous diagnostics				
Mitral insufficiency	11 (36.67%)	6 (33.33%)	5 (41.67%)	0.64
Tricuspid Insufficiency	11 (36.67%)	8 (44.44%)	3 (25%)	0.28
Mitral stenosis	6 (20%)	4 (22.22%)	2 (16.67%)	0.71
Medical therapy				
Beta-blockers	18 (60%)	11 (61.11%)	7 (58.33%)	0.88
Flecainide	9 (30%)	7 (38.89%)	2 (16.67%)	0.19
Amiodarone	4 (13.33%)	3 (16.67%)	1 (8.33%)	0.51

**Table 2:** Electrophysiological description of the simulations.

	Complete Cohort	AF-Freedom Group	AF Group	*p*-Value
Simulation characterization	30 patients	18 patients	12 patients	
Sustained simulations (%)	33.70 ± 17.73	33.00 ± 17.82	34.90 ± 17.63	0.79
PV attachment (%)	84.67 ± 6.83	86.20 ± 7.06	81.33 ± 5.97	0.10
Simulations presenting high entropy values in PV (%)	81.25	93.75	62.50	*p* < 0.001
Rotor distribution				
Right Atrium rotors	2.13 ± 0.38	2.22 ± 0.34	1.97 ± 0.40	0.09
Left Atrium rotors	2.97 ± 0.48	2.96 ± 0.35	2.97 ± 0.68	0.99
Left atrial appendage rotors	2.78 ± 3.45	3.52 ± 3.81	1.50 ± 2.37	0.14
Biomarkers from simulations				
ACM presenting high entropy areas in RA (%)	81.25	68.75	100	*p* < 0.001
Atrial Complexity Biomarker	1.53 ± 0.23	1.61 ± 0.21	1.40 ± 0.20	0.018
ACM presenting high entropy areas in RA (%)	81.25	68.75	100	*p* < 0.001

## Data Availability

Data will be available from the authors upon reasonable request.

## Supplementary Materials

The following are available online at https://www.mdpi.com/article/10.3390/biology10090838/s1, Figure S1: A. 200 × 200 simulation for Koivumaki ionic model B. 200 × 200 simulation for Alonso-Atienza automata model, Figure S2: Example of models after Jacquemet implementation. A. Complete Atria simulation for Koivumaki ionic model. B. Complete atria simulation for Alonso-Atienza automata model, Figure S3: A. Average number of sustained rotors siulations with respect to the number of initiated singularity points (SP). B. Percentage of sustained simulations with respect to number of rotors in pulmonary veins.
