# Supplementary material for: Personalized Evaluation of Atrial Complexity of Patients Undergoing Atrial Fibrillation Ablation: A Clinical Computational Study
Source: Biology (Basel). 2021 Aug 28;10(9):838. doi: 10.3390/biology10090838 (PMC8469429; doi:10.3390/biology10090838)
Supplement: Supplementary file 1 [file biology-10-00838-s001.zip › biology-1350926-supplementary.pdf]

Supplementary Material

Automata Models: Implementation and Electrophysiological Characteristics

We applied automata models consisting on probabilistic models in which each unit can present different discrete states that change according to simple rules as a function of the previous state and the state of the neighboring cells. Once the simulation is completed, each of the discrete states can be interpolated with an action potential to calibrate the model at electrophysiological level. A brief example on 2D planes is shown in Supplementary Figures 1 and 2, for both sinus rhythm and AF initiation respectively. As it can be observed, propagation along the plane is depicted in the figure along with the electrical activation. Koivumaki et al's model (Skibsbye et al., 2016) was simulated in a plane of 2 cm^2 side to simulate three coordinated impulses on an AF model, obtaining a conduction velocity of 45.45 cm/s and Action Potential Duration at 90% repolarization (APD90) of 193.95 ms. These parameters were later introduced in the automata model used for the study (Alonso-Atienza et al., 2005), to compare both simulations. As it can be observed, similar results were obtained for the propagation of the model and the electrical signal obtained from the automata model is similar to the one obtained in the detailed ionic model..

Automata Model: Alonso-Atienza Model Description

Alonso-Automata model (Alonso-Atienza et al., 2005) relies on three activation states that depend on the following modelling.

State 0 correspond to the resting phase, in which the tissue is relaxed and excitable. The excitation of the tissue, and therefore the depolarization phase, is modelled by the following equation:

$$P_j^{exc} = E * Q = E * \sum_{i \neq j} \frac{A_i}{D_{ij}^2}$$

Where E stands for excitability, A for activation and D corresponds to the distance matrix. Once the state is the activated one, corresponding to a cell that can is excited and can excite others, the behavior is modelled by the following equation:

$$APD = APD_{min} + APD_{max} * (1 - \exp(-0.5 * di))$$

Where the APD_{min} and APD_{max} correspond to the minimum and maximum values of the APD and the di corresponds to the diastolic interval. State 1 is modeled for the 10% time of the total APD. After this simulation time, State 2, corresponding to the refractory phase in which the tissue is exited but not able to activate the surrounding tissue, is modelled for 90% of the APD length (partial repolarization).

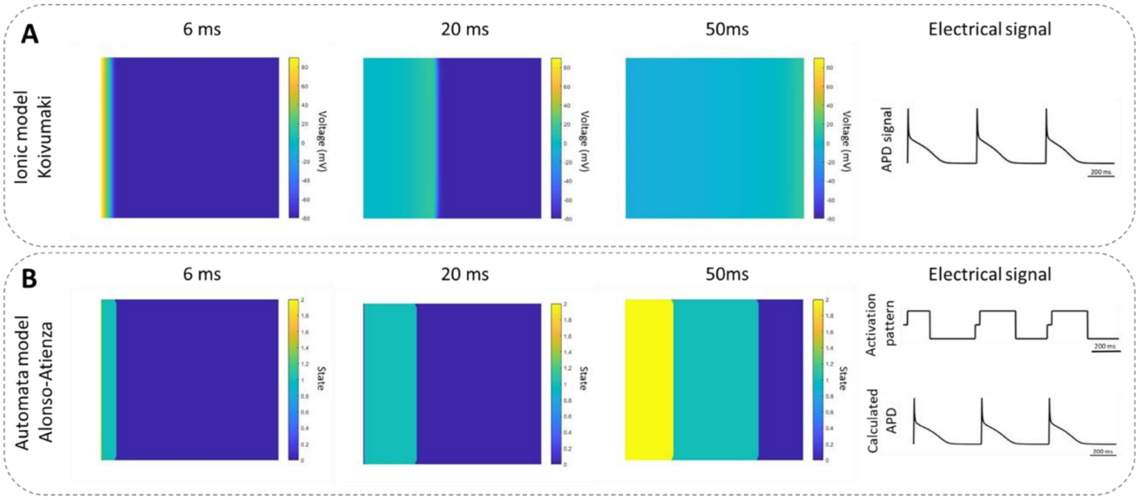

Sup. Figure S1. A. 200x200 simulation for Koivumaki ionic model B. 200x200 simulation for Alonso-Atienza automata model.

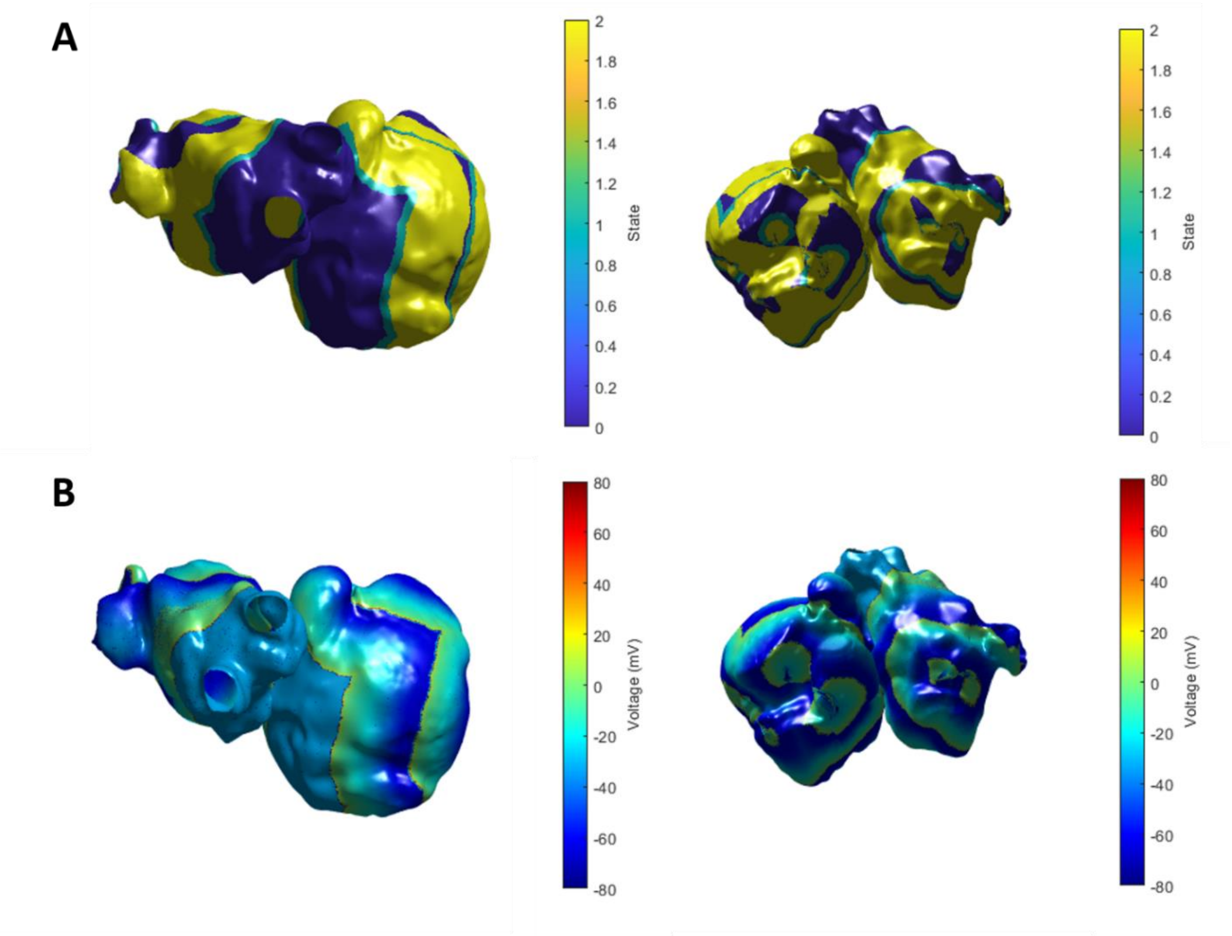

Sup Figure S2. Example of models after Jacquemet implementation A. Complete Atria simulation for Koivumaki ionic model. B. Complete atria simulation for Alonso-Atienza automata model.

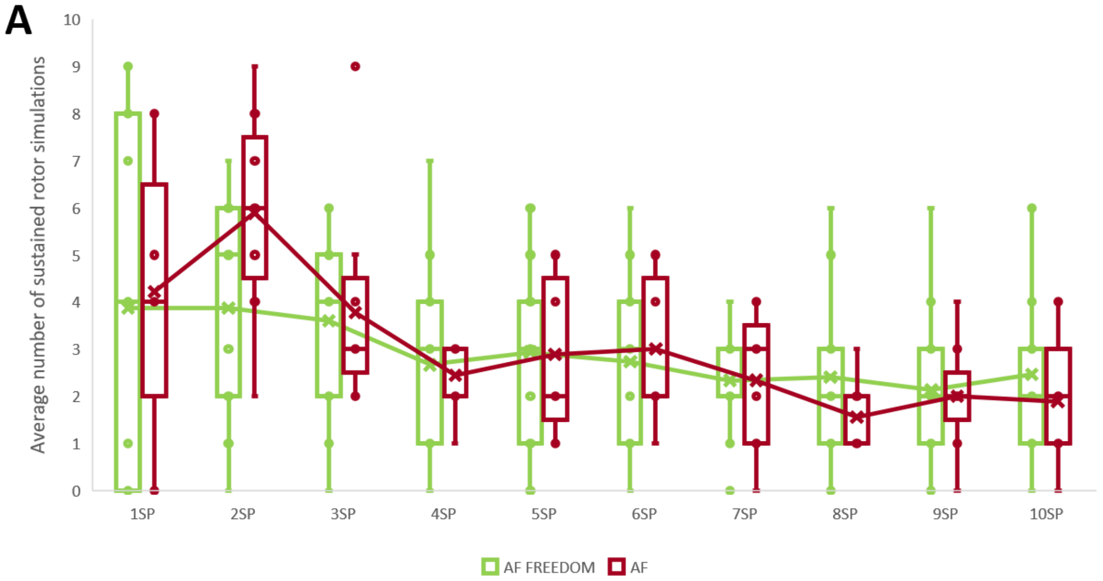

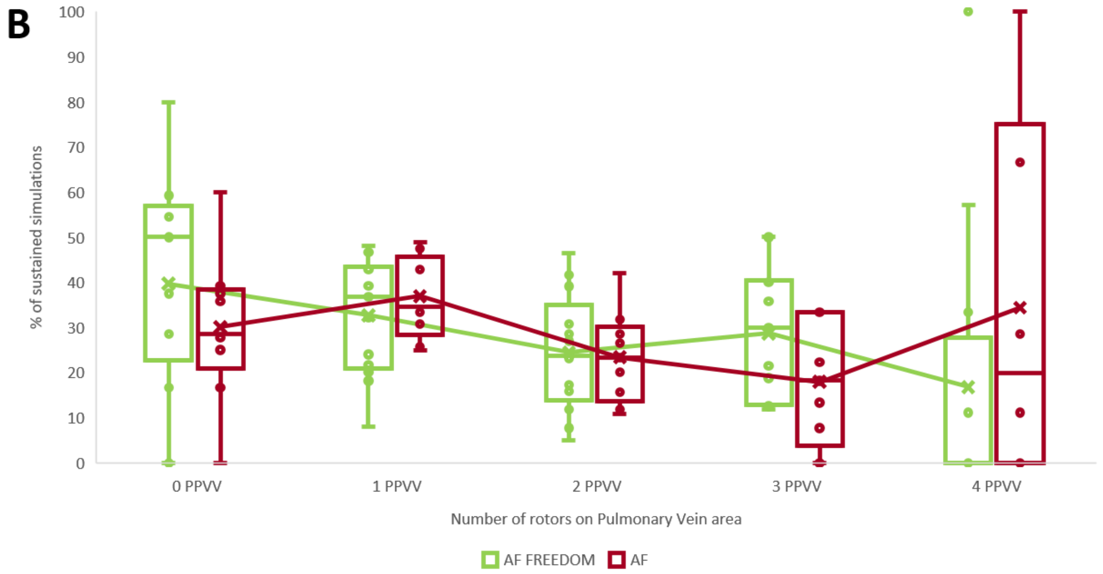

Sup. Figure S3. A. Average number of sustained rotor simulations with respect to the number of initiated singularity points (SP) B. Percentage of sustained simulations with respect to number of rotors in pulmonary veins.
